# Stunting predictors among children aged 0-24 months in Southeast Asia: a scoping review

**DOI:** 10.1590/0034-7167-2022-0625

**Published:** 2024-05-13

**Authors:** Via Eliadora Togatorop, Laili Rahayuwati, Raini Diah Susanti, Julianus Yudhistira Tan

**Affiliations:** I Universitas Padjadjaran, Faculty of Nursing. Bandung, Indonesia

**Keywords:** Children, Infant, Epidemiologic Factors, Southeast Asia, Stunting, Crianças, Lactente, Fatores Epidemiológicos, Sudeste da Ásia, Transtornos do Crescimento, Niños, Lactente, Factores Epidemiológicos, Sudeste de Asia, Trastornos del Crecimiento

## Abstract

**Objective:**

To identify predictors of stunting among children 0-24 months in Southeast Asia.

**Methods:**

This scoping review focused on articles with observational study design in English published from 2012 to 2023 from five international databases. The primary keyword used were: “stunting” OR “growth disorder” AND “newborn” AND “predict” AND “Southeast Asia”.

**Results:**

Of the 27 articles selected for the final analysis there are thirteen predictors of stunting in seven Southeast Asia countries. The thirteen predictors include the child, mother, home, inadequate complementary feeding, inadequate breastfeeding, inadequate care, poor quality foods, food and water safety, infection, political economy, health and healthcare, water, sanitation, and environment, and social culture factor.

**Conclusion:**

All these predictors can lead to stunting in Southeast Asia. To prevent it, health service providers and other related sectors need to carry out health promotion and health prevention according to the predictors found.

## RESUMO

**Objetivo:**

Identificar preditores de nanismo entre crianças de 0 a 24 meses no Sudeste Asiático.

**Métodos:**

Esta revisão de escopo se concentrou em artigos com desenho de estudo observacional em inglês publicados de 2012 a 2023 em cinco bases de dados internacionais. As palavras-chave primárias usadas foram: “stunting” (nanismo) OU “growth disorder” (distúrbio de crescimento) E “newborn” (recém-nascido) E “predict” (previsão) E “Southeast Asia” (Sudeste Asiático).

**Resultados:**

Dos 27 artigos selecionados para análise final, existem treze preditores de nanismo em sete países do Sudeste Asiático. Os treze preditores incluem a criança, a mãe, a casa, a alimentação complementar inadequada, a amamentação inadequada, os cuidados inadequados, os alimentos de má qualidade, a segurança alimentar e da água, a infeção, a economia política, a saúde e os cuidados de saúde, a água, o saneamento e o meio ambiente, e o fator cultura social.

**Conclusão:**

Todos estes preditores podem levar ao nanismo no Sudeste Asiático. Para preveni-lo, os prestadores de serviços de saúde e outros setores relacionados precisam realizar a promoção e a prevenção da saúde de acordo com os preditores encontrados.

## RESUMEN

**Objetivo:**

Identificar indicadores de desnutrición crónica entre niños de 0 a 24 meses en el Sudeste Asiático.

**Métodos:**

Esta revisión de alcance se centró en artículos con diseño de estudio observacional publicados en inglés, entre los años 2012 y 2023 de cinco bases de datos internacionales. Las principales palabras clave utilizadas fueron: “stunting (Desnutrición crónica)” o “growth disorder (Desorden del crecimiento)”, y “newborn (Recién nacido)”, y “predict (Predecir)”, y “Southeast Asia (Sudeste Asiático)”.

**Resultados:**

De los 27 artículos seleccionados para el análisis final, se encontraron trece indicadores que influyen en la desnutrición crónica en siete países del Sudeste Asiático. Los trece indicadores incluyen el niño, la madre, el hogar, la alimentación inadequada complementaria, la lactancia materna inadecuada, la atención inadecuada, los alimentos de mala calidad, la seguridad de los alimentos y el agua, la infección, la economía política, la salud y la asistencia sanitaria, el agua, el saneamiento y el medio ambiente, y por último el factor sociocultural.

**Conclusión:**

Todos estos indicadores pueden provocar desnutrición crónica en niños del Sudeste Asiático. Para poder prevenirlo los prestadores de servicios de salud y otros sectores relacionados, necesitan realizar actividades de promoción y prevención de la salud de acuerdo con los indicadores encontrados en este artículo.

## INTRODUCTION

Stunting is a manifestation of severe, irreversible physical, mental and cognitive impairment due to chronic malnutrition in early life, which becomes a global serious health problem, particularly in low-middle income countries and developing countries^(1)^. Based on global prevalence, there are as many as 22% or 149,2 million children under five with these cases, 35.2% in poor countries and 22.4% in developing countries, especially those spread across Asia, which has the largest stunting cases with a prevalence of 56%^(2)^. Although the level of stunting across the world is reduced, stunting in Southeast Asia is still high with a prevalence of stunting of 27.4%^(2)^.

Stunting has negative adverse effects, include short-term and long-term impact; short-term adverse effects cause impaired brain development, intelligence, physical growth disorders and metabolic disorders, and long-term effects are decreased cognitive abilities and learning achievement, decreased immunity and risk of other complications^(3)^. Moreover, one million deaths are attributable to stunting, especially in developing countries, and 28% of child mortality is caused by malnutrition^(4)^. Along with this prevalence, stunting can be caused by several factors, both direct and indirect, which include history of breastfeeding, low birth weight, short maternal posture, socioeconomic, environment and sanitation, and health services^(5)^.

Despite the various stunting interventions that have been provided by governments in Southeast Asia, the prevalence of stunting is still reported to be high and is also a major burden^(6)^. Tono added that this was happening because the existing stunting interventions were not in accordance with the determinants that should be addressed^(7)^. Although there are many studies that have found factors that cause stunting, the predictors of stunting in the golden period of children in Southeast Asia are still not known with certainty. Because information regarding predictors of stunting is very important to know in Southeast Asia, we conducted this scoping review to find out what are the predictors of stunting in children aged 0-24 months in Southeast Asia. It is hoped that the stunting predictors that we have summarized based on the WHO stunting conceptual framework can help determine better interventions and become material for further exploring various predictors for which the evidence is not clear enough.

## OBJECTIVE

To identify predictors of stunting among children 0-24 months in Southeast Asia.

## METHODS

This scoping review was conducted by following the guidelines of the Joanna Briggs Institute (JBI) methodology for Scoping reviews^(8)^ and reported using the Preferred Reporting Items for Systematic Review and Meta-Analyses (PRISMA) extension for Scoping Reviews^(9)^. The steps followed include: developing objectives and research questions, determining inclusion and exclusion criteria for data on evidence, planning search strategies, determining sources of evidence selection, data extraction, analysis of evidence, and presenting results^(8)^.

We used PCC (Population, Concept and Context) framework to formulate the review question. The population were children aged under 24 months. The concept focused on the predictors of stunting. The context of article was countries in Southeast Asia.

All articles included in this study were searched in five international databases on March 14, 2023. The five databases were MEDLINE/PubMed, Cumulative Index for Nursing and Allied Health Literature (CINAHL) plus with full text via EBSCOhost, Web of Science, Scopus, and Cochrane Library. The descriptors used contained the Medical Subject Heading (MeSH) and not limited to “stunting” OR “growth disorder” AND “infant” AND “epidemiologic factor” AND “Southeast Asia”. The full search strategy can be seen in Chart 1.

**Chart 1:** Search strategy used for study selection (Searched on 14-March-2023)

Search	Descriptors	Results
MEDLINE/PubMed		
#1	“Growth Disorders”[MeSH Terms] OR “stunting”[All Fields] OR “growth stunted”[All Fields] OR “growth disorder”[All Fields]	42,105
#2	“Infant”[MeSH Terms] OR “infant, newborn”[MeSH Terms] OR “Infant”[All Fields] OR “neonates”[All Fields] OR “newborn”[All Fields]	1,478,108
#3	“Epidemiologic Factors”[MeSH Terms] OR “epidemiologic factor”[All Fields] OR “determinants”[All Fields] OR “epidemiologic determinant”[All Fields] OR “Risk Factors”[MeSH Terms] OR “risk factor scores”[All Fields] OR “risk scores”[All Fields] OR “population at risk”[All Fields] OR “health correlates”[All Fields] OR “social risk factors”[All Fields] OR “predict^*^”[All Fields]	3,702,932
#4	“asia, southeastern”[MeSH Terms] OR “Southeast Asia”[All Fields] OR “Southeastern Asia”[All Fields]	121,111
#5	#1 AND #2 AND #3 AND #4	140
#6	#5 AND ((2012/1/1:2023/2/28[pdat]) AND (english[Filter]))	89
CINAHL Plus with Full Text via EBSCOhost		
#1	MW “stunting” OR TX “growth disorders” OR TX “chronic malnutrition” OR TX “stunted, disorder” OR TX “stunting” OR MW “growth disorder”	6,484
#2	MW (“infant” or “newborn” or “neonate”) OR TX (“infant” or “newborn infant” or “newborn” or “neonates”)	404,466
#3	MW (“epidemiologic determinant” or “risk factor” or “population at risk”) OR TX (“epidemiologic factors” or “determinants” or “risk factors” or “social risk factors” or “predict^*^” or “factors”)	2,254,678
#4	TX (“Vietnam” or “Timor-Leste” or “Laos” or “Philippines” or “Indonesia” or “Malaysia” or “Cambodia” or “Singapore” or “Thailand” or “Myanmar” or “Brunei”) OR TX (“Southeast asia” or “Southeastern asia”)	15,479
#5	#1 AND #2 AND #3 AND #4	50
#6	#5 Limiters - Published Date: 20120101-20230231; English Language; Peer Reviewed	41
Web of Science		
#1	(((((TS=(“stunting”)) OR TS=(“growth disorder”)) OR ALL=(“stunting”)) OR ALL=(“chronic malnutrition”)) OR ALL=(“disorder, stunted”)) OR ALL=(“stunted”)	15,028
#2	(((((((((TS=(“epidemiologic factor”)) OR ALL=(“predict^*^”)) OR ALL=(“determinants”)) OR ALL=(“epidemiologic determinant”)) OR TS=(“risk factor”)) OR All=(“risk factor”)) OR ALL=(“social risk factor”)) OR ALL=(“risk scores”)) OR ALL=(“risk factor scores”)) OR ALL=(“population at risk”)	5,010,207
#3	((((TS=(“infant”)) OR TS=(“newborn infant”)) OR ALL=(“newborn”)) OR ALL=(“infant”) OR ALL=(“neonates”)) OR TS=(“neonate”)	434,720
#4	#1 AND #2 AND #3	397
#5	#4 AND English (Languages) AND INDONESIA OR CAMBODIA OR THAILAND OR PHILIPPINES OR MYANMAR OR MALAYSIA OR VIETNAM OR SINGAPORE (Countries/Regions)	31
Scopus		
#1	TITLE-ABS-KEY (“stunting” OR “growth disorder”) OR ALL (“stunting” OR “growth disorder” OR “stunted, disorder” OR “chronic malnutrition”)	59,280
#2	TITLE-ABS-KEY (“infant” OR “newborn”) OR ALL (“infant” OR “newborn” OR “newborn infant” OR “neonates”)	3,018,229
#3	TITLE-ABS-KEY (“epidemiologic factor” OR “determinant, factor” OR “determinant” OR “risk factor”) OR ALL (“determinant factor” OR “epidemiologic factor” OR “predict^*^” OR “determinant” OR “factor” OR “risk factor” OR “social risk factor” OR “risk scores” OR “risk factor scores”)	28,283,038
#4	TITLE-ABS-KEY (“Southeast Asian” OR “South-eastern Asia”) OR ALL (“Southeast Asia” OR “Southeast Asian” OR “Southeastern Asia” OR “South-eastern Asia”)	389,920
#5	#1 AND #2 AND #3 AND #4	746
#6	#5 AND ( LIMIT-TO ( LANGUAGE , “english” ) ) AND ( LIMIT-TO ( PUBYEAR , 2023 ) OR LIMIT-TO ( PUBYEAR , 2022 ) OR LIMIT-TO ( PUBYEAR , 2021 ) OR LIMIT-TO ( PUBYEAR , 2020 ) OR LIMIT-TO ( PUBYEAR , 2019 ) OR LIMIT-TO ( PUBYEAR , 2018 ) OR LIMIT-TO ( PUBYEAR , 2017 ) OR LIMIT-TO ( PUBYEAR , 2016 ) OR LIMIT-TO ( PUBYEAR , 2015 ) OR LIMIT-TO ( PUBYEAR , 2014 ) OR LIMIT-TO ( PUBYEAR , 2013 ) OR LIMIT-TO ( PUBYEAR , 2012 ) )	577
Cochrane Library		
#1	(“stunting”):ti,ab,kw OR (“disorder, growth”) OR (“disorder, stunted”) OR (“chronic malnutrition”) OR (“stunting”)	1,342
#2	MeSH descriptor: [Growth Disorders] explode all trees	1,307
#3	(“infant”):ti,ab,kw OR (“newborn infant”):ti,ab,kw OR (“newborn”) OR (“infant”) OR (“neonates”)	88,412
#4	(neonates):ti,ab,kw	9,027
#5	MeSH descriptor: [Infant] explode all trees	39,686
#6	MeSH descriptor: [Infant, Newborn] explode all trees	20,292
#7	(“epidemiologic factors”):ti,ab,kw OR (“epidemiologic determinant”):ti,ab,kw OR (“prediction”) OR (“determinant”) OR (“risk factors”):ti,ab,kw	80,650
#8	(“social risk factor”) OR (“risk scores”) OR (“risk scores scores”) OR (“population at risk”)	2,090
#9	MeSH descriptor: [Epidemiologic Factors] explode all trees	58,374
#10	MeSH descriptor: [Risk Factors] explode all trees	32,696
#11	(“Southeast Asian”):ti,ab,kw OR (“Southeastern Asia”):ti,ab,kw OR (“Southeast Asia”)	663
#12	MeSH descriptor: [Asia, Southeastern] explode all trees	3,501
#13	(#1 or #2) AND (#3 or #4 or #5 or #6) AND (#7 or #8 or #9 or #10) AND (#11 or #12) with Publication Year from 2012 to 2023, with Cochrane Library publication date Between Jan 2012 and Feb 2023, in Trials	9

*MeSH-medical subject heading; pdat-publication date; TX-all text; MW-word in subject heading; PUBYEAR-publication year.*

To clearly identify the most recent and specific predictors of stunting in the Southeast Asia region, there were two search limitations that we considered important to be applied. First, we only included peer-reviewed articles in English language and published from January 2012 to February 2023. Second, we limited the geographic location of the study to Southeast Asia only. In addition, we included only original studies with observational designs and secondary data analysis. The secondary data analysis was included because the nature of the study is basically observational and retrospective. All relevant full text articles that were not found after being searched by the authors, including asking as to the author of the article, were excluded from the review.

Following the search, all identified articles were collated and uploaded into a website application named Rayyan for review^(10)^, to immediately proceed with the removal of duplicates and screening. Titles and abstracts were also immediately screened by the first and fourth authors, thereby obtaining relevant articles for review. Potentially relevant articles were retrieved in full text and assessed in detail based on inclusion criteria by all authors independently. The article selection process was recorded and reported in the PRISMA 2020 flow diagram^(11)^. Any disagreements that arose between authors at each stage of the selection process were resolved through discussion.

The article data were extracted by all authors independently using the JBI data extraction instrument which had been developed according to the purpose of the review. The data extraction covered the characteristics of article, study objectives, population, and outcomes. For the characteristics of the articles, we extracted the authors, year of publication, country of origin, and study design. For the population, we extracted the numbers and the main criteria of the sample. Finally, for the outcome, we only extracted the predictors of stunting found in children aged 0-24 months, including their statistical values. Any disagreements that arose in the data extraction process were resolved through discussion.

According to the guidelines used^(8)^, this scoping review presents two tables of the data extraction results, which were agreed upon by all authors and provide a narrative summary of the findings of the articles reviewed. The first table describes the characteristics of the articles and the predictors of stunting found. The second table provides a summary of the predictors of stunting classified by factors from the WHO stunting conceptual framework^(12)^. The narrative summary focused on describing the number of existing predictors of stunting and the most common predictors of stunting in Southeast Asia. The chart’s template also presents the checklist for review articles.

## RESULTS

**Figure 1:**
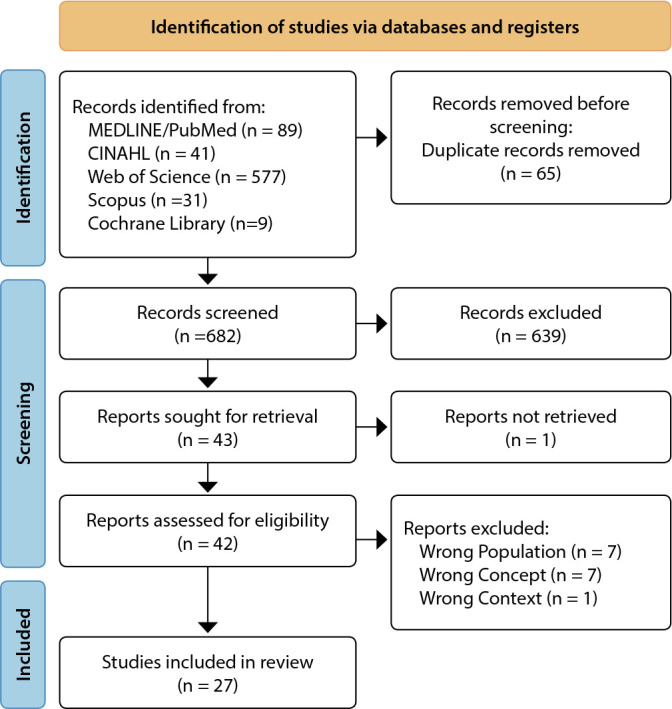
Process of identification, screening, and selection of studies

The results of identification and screening of 747 articles showed that there were 27 articles that met the inclusion criteria. Another 720 articles were excluded following the PRISMA-ScR flow diagram (see Figure 1). Of 27 studies, the predictors of stunting have been widely studied in Indonesia^(13-23)^. Other Southeast Asian countries that have also studied the predictors of stunting are the Philippines^(24-29)^, Cambodia^(30-32)^, Vietnam^(33-34)^, Myanmar^(35-36)^, Malaysia^(37-38)^, and Brunei Darussalam^(39)^. From our defined population, few studies appear to have specifically studied the predictors of stunting in children aged 6-24 months only^(19-20,27-28,31-33,35-36)^ and/or have also examined the caregiver or household of children^(15,24-25,29-30,34)^. Regarding the study designs used, secondary data analysis (14/27 studies) is the most widely used study design. Others were identified using case-control (1/27 studies), cohort (5/27 studies) and cross-sectional (7/27 studies) (see Chart 2).

**Chart 2:** Synthesis of the studies selected for the scope review

Authors /Year	Design	Sample/ Country	Study Objectives	Findings
Torlesse et al/ 2016^(13)^	Cross-sectional	1,366 children aged 0-23 months/ Indonesia	To identify factors associated with stunting in children aged 0-23 months.	Age of child: 12-23 months (aOR 4.40)^^***^^ Low household wealth (aOR 2.30)^^**^^ Male gender (aOR 1.45)^^**^^
Sari et al/2021^(14)^	Secondary data analysis	756 infants/ Indonesia	To determine the relationship between physical factors of parents and children with the incidence of stunting at birth	Stunting at birth Age of mother at first pregnancy^^***^^: 15-17 y (aOR 0.37), 18-19 y (aOR 0.37), 20-24 y (aOR 1.34), ≥25 y (aOR 0.80) Number of parities^^***^^: one parity (aOR 2.31), two parities (aOR 2.04), three parities (aOR 1.70) Preterm birth (aOR 2.12)^^***^^ Parental height with mother <145 cm and father <161.9 cm (aOR 5.93)^^***^^ Age of parents: either parent 20-35 years (aOR 1.50), both parent <20 or >35 years (aOR 2.37)^^***^^
Hadi et al/2021^(15)^	Secondary data analysis	408 caregivers of children aged 6-24 months/ Indonesia	To examine the role of exclusive BF in reducing stunting prevalence among children <2 years old attributable to low HH in a low income Indonesian population	Male gender (aOR 1.51)^+^ Exclusive BF (aOR 0.82)^+^ Age of child: <12 months (aOR 0.20)^+^ Mother as caregiver (aOR 0.38)^+^ Maternal employment status: unemployed (aOR 8.40), labor/entrepreneur (aOR 4.52), farmer/breeder (aOR 11.74)^+^ Monthly HH expenditure: <USD 15 (aOR 1.03), USD 15 to <25 (aOR 2.00), USD 25 to <37 (aOR 2.28), USD 37 to <54 (aOR 1.20), >USD 54 (aOR 1)^+^
Gunardi et al/2018^(16)^	Cohort	160 children aged <24 months/ Indonesia	To identify parental socio-demographic risk factors of declined linear growth in children under 2 years.	Maternal education ≤9 years (RR 2.60)^^*^^ Working mother (RR 0.17)^^*^^
Sartika et al/2021^(17)^	Cross-sectional	559 infants/ Indonesia	To determine important factors of childhood stunting with a focus on maternal and child nutrition, and pre-postnatal determinants	*Adjusted with LBW* LBW (aOR 4.119)^^**^^ Diarrhea (aOR 3.227)^^***^^ Preterm birth (aOR 5.10)^^*^^ Incomplete immunization (aOR 2.43)^^*^^
Titaley et al/2019^(18)^	Secondary data analysis	24,657 children aged <24 months/ Indonesia	To examine the determinants of stunting in children aged <24 months using the 2013 Indonesia Basic Health Survey data	>3 HH members who aged under 5 years (aOR 1.33)^^*^^ 5-7 HH members (aOR 1.1)^^**^^ HH wealth: poorest (aOR 1.74)^^***^^ 1-3 times prenatal care visitation (aOR 1.22)^^**^^ Male gender (aOR 1.33)^^***^^ Age of child: 12-23 months (aOR 1.89)^^***^^ LBW (aOR 2.55)^^***^^
Nurhayati et al/2020^(19)^	Case-control	158 children aged 6-23 months/ Indonesia	To examine the relationship between dietary diversity and vitamin D intake with stunting in children aged 6-23 months	Age of child: 18-23 months (OR 3.80)^^*^^ BL ≥48 cm (OR 0.36)^^*^^ Poor micronutrient quality: vitamin D (OR 5.18)^^*^^ Dietary diversity with ≥4 food groups (OR 0.17)^^*^^
Madinar et al/2021^(20)^	Cross-sectional	231 children aged 6-23 months/ Indonesia	To determine the factors related to stunting among children aged 6-23 months.	MAD is not met (OR 3.29)^^*^^ Short BL (OR 0.471)^^*^^ Family income level (OR 0.387)^^**^^
Utami et al/2018^(21)^	Cohort	320 children aged <23 months/ Indonesia	To determine the major factors of stunting among children aged 0-23 months	BW <3000 gr (HR 1.847)^^***^^ BL <48 cm (HR 1.567)^^*^^ Maternal height <150 cm (HR 1.436)^^*^^
Hanifah et al/2018^(22)^	Secondary data analysis	4,677 children aged 0-23 months in 2000, 2007, and 2014/ Indonesia	To determine the trend of stunting incidence and associated factors in 2000 and 2014	In 2000: Age of child^^***^^ LBW^^*^^ Maternal education status^^**^^ Completeness of prenatal care^^**^^ Inadequate sanitation^^***^^ Completeness of immunization^^*^^ In 2014: Age of child^^***^^ BW^^***^^ Provision of prelacteal^^***^^ Ownership of Mother and Child Health Book/Card ^^**^^ Inadequate sanitation^^*^^
Diana et al/2021^(23)^	Cohort	230 healthy full term, breastfed infants/ Indonesia	To identify factors influencing the linear growth children in rural setting	Stunting in children aged 6 to 12 months Maternal height (SMD 0.21)^^**^^ Improved drinking water source (SMD -0.31)^^*^^
Rohner et al/2013^(24)^	Cross-sectional	1,777 HH with children aged 6-23 months/ Philippines	To determine factor associated with the nutritional status of children aged 6-23 months living in urban areas of the Philippines.	HH with poorest socioeconomic status (aOR 4.8)^^***^^ Using wood or coal as fuel for cooking (aOR 1.5)^^*^^ Age of child (aOR 1.08)^^***^^ Not consuming daily or weekly multivitamin (aOR 1.5)^^*^^
Maravilla et al/2020^(25)^	Cohort	1,033 singleton birth mothers aged 14-24 years/ Philippines	To measure the magnitude of the association between repeated pregnancy in adolescent mother and stunting incidence in children aged 12 and 24 months	Stunting in children aged 12 months Repeated pregnancy (aOR 1.40)^^***^^ LBW (aOR 2.52)^^***^^ BF within 24 hours after delivery (aOR 1.42)^^*^^ BF until 12 months (aOR 0.34)^^*^^ Solid foods within 6-8 months of age (aOR 0.31)^^*^^
Hinojosa et al/2021^(26)^	Secondary data analysis	5,254 children aged 0-23 months/ Philippines	To determine the relationship between maternal characteristics and nutritional status of children aged 0-23 months based on their length per height for age	College level maternal education (OR 0.39)^^*^^ BF for 1-6 mo (OR 0.58) and >1 year (OR 2.64)^^***^^ Age-appropriate BF (OR 0.63)^^*^^ Third semester of pregnancy age: (OR 2.95)^^**^^ Having tetanus toxoid vaccine at prenatal care (OR 0.67)^^*^^ Having ultrasound assessment at prenatal care (OR 0.71)^^*^^
Guirindola et al/2021^(27)^	Secondary data analysis	2,275 children aged 6-23 months/ Philippines	To identify the drivers of stunting in young Filipino children aged 6-23 months	Age of child: 12-23 months (RR 3.04)^^**^^ Male gender (RR 1.99)^^**^^ LBW (RR 2.19)^^**^^ Age of mother: <20 years (RR 1.90)^^*^^ Maternal height <151 cm (RR 2.33)^^**^^ Maternal education level is primary school and less (RR 1.59)^^*^^ Wealth status is poor (RR 1.69)^^*^^ Not given prelacteal (RR 0.67)^^*^^ MMF is not met (RR 1.82)^^*^^
Ulep et al/2022^(28)^	Secondary data analysis	1,881 children aged 6-23 months/ Philippines	To determine the magnitude of socioeconomic inequality in stunting among 6-23 months Filipino children and to decompose the inequality into maternal, health and nutrition and socio-demographic factors	Male gender (β=-0.11)^^*^^ Maternal height (β=-0.02)^^*^^ Divorced marital status (β=0.17)^^*^^ BMI^^*^^: normal (β=-0.11), overweight (β=-0.16), obese (β=-0.20) MMF (β=-0.15)^^*^^ DDS (β=-0.02)^^*^^ High quality of prenatal care (β=-0.06)^^*^^ Iron supplementation in children (β=-0.06)^^*^^
Blake et al/2016^(29)^	Secondary data analysis	357 mother-infant pairs/ Philippines	To compare rates of stunting, wasting and underweight among infants with LBW, SGA, non-LBW, and non-SGA at one, six and 12 months of age	Male gender (OR 1.78)^^**^^ Gestational age (OR 0.69)^^***^^ LBW (OR 3.82)^^***^^ SGA (OR 2.98)^^***^^ Non-exclusive BF (OR 2.30)^^**^^ Maternal height at first semester of pregnancy (OR 0.95)^^**^^ Maternal weight at first semester of pregnancy (OR 0.97)^^*^^
Kpewou et al/2020^(30)^	Secondary data analysis	779 mothers-infant pairs/ Cambodia	To describe the effect of mother’s nutritional status during pregnancy on early child growth	MUAC at pregnancy <23 cm (OR 1.676)^^*^^ Maternal education level is secondary school and above (aOR 0.46)^^*^^ Male gender (aOR 1.57) ^^*^^ HH wealth index: richest (OR 0.41)^^*^^
Laillou et al/2020^(31)^	Secondary data analysis	1,938 children aged 6-23.9 months/ Cambodia	To explore the factors that contribute to stunting and wasting in children	Percentage to predict stunting. Low wealth quintile (21.4-45.2%) Mother without formal education (12.2-30.6%) Low child feeding index score (15.4-22.3%) No access to vitamin A supplementation (5.4-15.8%) No birth certificate (2.5-14.1%) Access to non-improved sanitation facility (1.9-8.3%) Inadequate treatment of water (1.2-10.2%) Access to non-improved drinking water (5.3-11.3%)
Harvey et al/2022^(32)^	Secondary data analysis	1,365 children aged 6-24 months/ Cambodia	To identify and investigate the complex pathways to stunting among children aged 6-24 months cioeconomic factors	Dietary diversity (β=1, direct effect)^^*^^ HH wealth (β=0.712; direct effect, β=0.275; indirect effect)^^**^^ BF (β=-0.948; direct effect)^^***^^ Maternal education (β=0.051; indirect effect)^^***^^ Maternal employment (high level of participation; β=0.414; indirect effect)^^***^^
Beal et al/2019^(33)^	Secondary data analysis	30,771 children aged 6-23 months/ Vietnam	To identify the stunting determinants among children aged 6-23 months.	Age of child, years (aRR 2.49)^^***^^ Not consuming BF (aRR 1.44)^^**^^ Age-appropriate BF (aRR 0.65)^^***^^ Male gender (aRR 1.35)^^***^^ Ethnic minority (aRR 1.31)^^***^^ LBW (aRR 1.75)^^***^^ Not given vitamin A in past 6 months (aRR 1.13)^^**^^ Maternal age <18 years (aRR 1.09)^^*^^ Maternal height ^^***^^:<145 cm (aRR 2.04), 145-149cm (aRR 1.62) Maternal BMI <18.5 (aRR 1.20)^^***^^ Maternal education^^***^^: no formal education (aRR 1.77), primary level (aRR 1.77), secondary level (aRR 1.57), high school level (aRR 1.38) Maternal employment: farmer (aRR 1.09)^^*^^ No perinatal iron or iron-folate tablet (aRR 1.15)^^**^^ HH with more than 2 children (aRR 1.10)^^*^^ Not using iodized salt (aRR 1.11)^^*^^ Residence type^^*^^: rural (aRR 1.14), poor commune (aRR 1.11)
Young et al/2018^(34)^	Secondary data analysis	1.409 mothers with at least one ultrasound before 30 weeks/ Vietnam	To examine associations between preconception maternal nutritional status and linear growth children across the first 1000 days.	Adjusted with BMI <18 kg/m^2.^ Maternal height <150cm (aRR 2.10)^^***^^ BMI at preconception (aRR 1.39)^^**^^ Age of child (aRR 0.86)^^*^^ Male gender (aRR 1.29)^^*^^ Maternal education level^^**^^: primary (aRR 2.30), secondary (aRR 2.30)
Mya et al/2019^(35)^	Secondary data analysis	1,222 last children living with their mother and aged 6-23 months/ Myanmar	To explore the relationship between IYCF practices and nutritional status of children age 6-23 months	Currently breastfed (aOR 0.51) ^^*^^ Female gender (aOR 0.46)^^***^^ Birth size below average (aOR 2.38)^^***^^ Maternal height^^*^^: 150-159 cm (aOR 0.42), ≥160 cm (aOR 0.41) Working mother (aOR 1.97)^^**^^ Rural residence (aOR 2.08)^^*^^
Phu et al/2019^(36)^	Cross-sectional	216 children aged 6-24 months/ Myanmar	To assess the influence of dependent care to stunting.	Feeding index^^***^^: low (OR 15.45), medium (OR 4.55)
Ahmad et al/2021^(37)^	Cohort	344 infants aged ≥3 months/ Malaysia	To identify the relationship between SGA and its effects on the growth patterns and nutritional status of infants.	Male gender (aOR 3.51 at one month; aOR 9.20 at six months; aOR 5.66 at 12 months)^^***^^ BW <2500 gr (aOR 25.47 at one month; aOR 6.62 at six months)^^*^^
Masroor et al/2014^(38)^	Cross-sectional	910 children aged <24 months/ Malaysia	To assess the nutritional status of children aged 0-24 months	Residence^^**^^ Age of child^^**^^ Gender^^**^^ Number of HH members^^**^^ BW^^***^^ Ethnic^^*^^ Income per capita^^*^^
Boylan et al/2017^(39)^	Cross-sectional	396 children aged <24 months/ Brunei Darussalam	To determine the prevalence and factors associated with stunting in children aged 0-24 months	Male gender (aOR 2.48)^^***^^ Preterm birth (aOR 2.14)^^*^^ BW <2500 gr (aOR 2.99)^^**^^

HR-hazard ratio; RR-relative risk; aRR-adjusted relative risk; OR-odds ratio; aOR-adjusted odds ratio; BF-breastfeeding; BL-birth length; BMI-body mass index; ; BW-birth weight; HH-household; IYCF-infant and young child feeding; LBW-low birth weight; MAD-minimum acceptable diet; MMF-minimum meal frequency; SGA-small for gestational age; SMD-standardized mean difference; WASH-water, sanitation and hygiene; ^^*^^ p value<0.05; ^^**^^ p value<0.01; ^^***^^ p value<0.001;

+ p value<0.1

By adapting the WHO stunting conceptual framework^(12)^, there are 13 factors of stunting in Southeast Asia (see Chart 3). Those factors are the child, mother, home, inadequate complementary feeding, inadequate breastfeeding, inadequate care, poor quality foods, food and water safety, infection, political economy, health and healthcare, water, sanitation, and environment, and social culture factor. Each of these factors has its own predictor of stunting, in which the total predictors are 34. Based on our summary, the predictors of stunting in Southeast Asia are mostly categorized in the mother factor (7/34 predictors).

**Chart 3:** Predictors of stunting according to WHO stunting conceptual framework

Factors	Predictors
The Child	Small for gestational age^^*^(29)^ Birth weight^^*^(17-18,21-22,25,27,29,33,37-39)^ Gender^^*^(13,15,18,27-30,33-35,37-39)^ Age^^*^(13,15,18-19,22,24,27,33-34,38)^ Birth lengt^h^*^(19-21,35^)
The Mother	Age at first pregnancy^^*^(14)^ Parity^^*^(14,25)^ Preterm birth^(14,17,29,39)^ Short maternal stature^(14,21,23,27-29,33-35)^ Poor nutrition during preconception and pregnancy^(28-30,33,34)^ Infection^(26)^ Prenatal car^e^*^(18,22,26^)
The home	Low wealth and socioeconomic status^(13,15,18,20,24,27,30-32)^ Inadequate sanitation and water supply^(22-23,31)^ Low caregiver education^(16,22,26-27,30-34)^ Number of household member^^*^(18,33,38)^ Age of parents^^*^(14,33)^
Inadequate complementary feeding	Infrequent feeding^(20,27-28,31,36)^ Complementary feeding initiation^^*^(25)^
Inadequate breastfeeding	Delay initiation^(25)^ Non-exclusive breastfeeding^(15,22,27,29)^ Early cessation of breastfeeding^(25-26,33,35)^
Poor quality foods	Poor micronutrient quality^(19,24,28,31,33)^ Low dietary diversity^(19-20,28,31-32,36)^
Food and water safety	Unsafe preparation of foods^(24)^
Infection	Enteric infection: Diarrheal disease^(17)^
Inadequate care	Poor care practices^(17,22,26)^
Political economy	Poverty, income, and wealth^(38)^ Employment^(15-16,32-33,35)^
Health and healthcare	Qualified healthcare providers^(28)^ Health care systems and policies^(22,31)^
Water, sanitation, and environment	Population density^(33,35,38)^
Society and culture	Child caregiver: mother^(15)^ Ethnic^^*^(33,38)^ Women’s status^(28)^

^^*^^ Newly added predictors.

Of the 34 predictors, there are 13 new predictors of stunting, which previously did not exist in the WHO stunting conceptual framework^(12)^. Specifically, from the results of categorizing these new predictors, it appears that five of them are highly related to the child’s condition. Thus, the child factor which was previously stated to be related to the consequences of stunting, is considered as one of the causal factors of stunting itself in Southeast Asia. Then, the remaining seven predictors are categorized into three factors related to the household (mother, home, and complementary feeding) and one factor related to the community (society and culture).

Based on the reported measures of effect, age of child (at 12-23 months or annually)^(27,33)^, maternal education (≤9 years or only primary or secondary)^(16,34)^, low birth weight^(27)^, and short maternal stature (<151 cm)^(27,34)^ are the predictors with the largest relative ratio, which increases the risk of stunting in Southeast Asian countries by up to two times. Then, regarding the odds of stunting in children aged 0-24 months, the odds increase up to five times in children who are male (at six months; aOR 9.20, at 12 months; aOR 5.66)^(37)^, have low birth weight (<2500 gr)^(37)^, are born prematurely (aOR 5.10)^(17)^, have short parental height (mother <145 cm and father <161.9 cm; aOR 5.93)^(14)^, have unemployed mothers (aOR 8.40)^(15)^ or whose mother work as farmer/breeder (aOR 11.74)^(15)^. Specifically for low birth weight, it was identified that the odds of a child becoming stunted increased by more than 25 times in the first month (aOR 25.47) and six times in the sixth month (aOR 6.62)^(37)^.

## DISCUSSION

Stunting is a type of malnutrition in children. Apart from having a negative impact on the growth and development of children, the quality of life and the future of children is also at great risk of decreasing due to stunting. Especially in Southeast Asia, which has the second highest prevalence of stunting among other subregions in Asia, there are 13 factors which consist of 34 predictors of stunting. Specifically, nine of these causal factors are closely related to the household and the rest are related to the community. However, because to the best of our knowledge, this review is the first to discuss predictors of stunting in children 0-24 months in Southeast Asia, further studies are still urgently needed in order to better understand predictors of stunting in all Southeast Asian countries.

### The Mother

Aside from being a causal factor that has the most predictors of stunting, the mother’s factor is very important to be targeted for the stunting prevention interventions. Of the seven predictors found, the majority of them were closely related to preconception and pregnancy conditions. Given the magnitude of the odds and risks of stunting from the reported predictors, it seems that all of these predictors may have to be addressed from the preconception period with a focus on nutrition and the mother’s readiness to care for her child since pregnancy. Particularly in predictors of prenatal care, nutrition, infection, and preterm birth, perhaps the management should be repeated and more focused during the pregnancy. This is because it is proven that the low number of prenatal care visits and not receiving Fe tablets during pregnancy increases the odds and risk of stunting by 22%^(18)^ and 15%^(33)^, respectively. Meanwhile, for the short maternal stature, because the high risk of stunting given by this predictor may be influenced by genetic mechanisms^(40)^ and intergenerational effect^(41)^, this predictor may be difficult to overcome.

Furthermore, of all the predictors, only the predictors of age at first pregnancy and infection were proven to reduce the odds of stunting. It was reported that giving the tetanus vaccine in prenatal care could reduce the odds of stunting by 33%^(26)^, and, interestingly, the younger (15-19 years) or older (≥25 years) age at first pregnancy was shown to reduce the odds of stunting events by 63% and 20% respectively^(14)^. Regarding the age at first pregnancy, mothers who are ≥25 years old are more likely to live with guaranteed conditions, both in terms of sanitation, socioeconomic, education or urban living areas^(42)^, but for the younger age range, further studies may be needed to confirm the results.

### The Child

In the child factor, even though this factor was not initially determined to be a causal factor for stunting, all of its predictors, except for the predictor of SGA, are supported by many studies. Gender, age, and birth weight of the child are predictors with the most supporting studies. However, specifically the age and gender of the child, due to their nature, this predictor which has also been shown to increase the risk of stunting up to three times (RR 3.04) and 99% (RR 1.99) respectively^(27)^, cannot be modified directly.

Apart from the fact that from 12 months to 24 months of age there will be a difference in body length between normal and LBW children^(43)^, as they get older, they may be exposed to many diseases and poor environmental conditions^(44)^. Furthermore, it is also known that the nutrition and diet that children need must be more adapted to their age, especially in relation to the child feeding practices^(44)^. Thus, we consider that, in order to overcome the increased odds or risk of stunting due to the child’s age, the direct cause must be clearly known.

Regarding birth weight, we note that this predictor provides the greatest odds of stunting compared to other predictors. Ahmad et al. analyzed that children with LBW could have a 25.47 times the odds of stunting in the first month of life^(37)^. Even though there will be a decrease in the odds as the child gets older, in the sixth month of life, the odds of stunting are still 6.62 times^(37)^. Guirindola et al. also explained that, based on the risk ratio, children with low birth weight were proven to have an increased risk of stunting by 2.19 times^(27)^. As with SGA, which also doubles the odds of stunting^(29)^, we think that the magnitude of all the odds and the risk of stunting is caused by the high vulnerability of children to contracting diseases and infections, as well as the immaturity of several children’s organs to be able to digest food properly. Therefore, in order to prevent the odds and risks posed by these predictors, stunting interventions may indeed have to be focused on nutrition during the golden period or the mother during her pregnancy, so that the child is not born with a low birth weight.

### The Home

As one of the causal factors of stunting, we consider that the home is a fairly comprehensive factor. This is because the predictors in this factor appear to be not only causal, but also contextual in a small scope (i.e. the child’s household). These contextual predictors include wealth and socioeconomic status and sanitation and water supply. Even though it was explained that wealth and socioeconomic status increased the risk of stunting by 69%^(27)^, another study also showed that this predictor also had an indirect effect on the incidence of stunting by 28%^(32)^. Regarding the indirect effect, dietary diversity and breastfeeding are the two other predictors that have been shown to significantly mediate the effect^(32)^. Thus, the predictors in this factor may not always directly cause stunting in children 0-24 months, but it can make other predictors to be media in causing stunting.

Of all the existing predictors, the education of the caregiver, which is the mother, is the predictor that gives the greatest risk of stunting in children 0-24 months. Literature shows that the risk of stunting is significantly greater when the mother has only primary education or even less^(16,27,33-34)^, but when the mother has a secondary education level or even college it can reduce the risk or odds of stunting in children^(26,30)^. With regard to those findings, we consider that the education level of mothers seems to have an indirect effect on stunting^(32)^, in which mothers will only be able to provide good quality care to their children, even from pregnancy, if their knowledge is high enough and/ or they understand their child’s nutritional needs.

### Inadequate Breastfeeding and Inadequate Complementary Feeding

Based on the studies reviewed, inadequate breastfeeding and inadequate complementary feeding are two other causal factors of stunting which are also especially important to be promptly managed in Southeast Asia. Although the number of predictors of these two factors is not as great as the previous factors, these predictors are closely related to child nutrition in the golden period. Thus, we suspect that any problems that arise in these two factors seem to be able to directly cause children to become stunted^(32)^.

Of the three predictors of breastfeeding factor, we note that early cessation from breastfeeding provides the greatest increase in the risk of stunting, which is up to 44%^(33)^. In fact, if breastfeeding is weaned properly, the odds and risk of stunting can be reduced by 37% and 35%, respectively. It was also explained that, apart from helping the growth and development^(26,29,45)^, by weaning only when the child is two years old, the child will be protected from many infectious diseases, and even increase the chances of life^(46)^. However, for proper weaning to occur, two other predictors of breastfeeding, which are the initiation and exclusive breastfeeding, must also be considered and introduced to the mother.

Regarding the complementary feeding factor, including the one we just added, there are only two predictors of this factor in Southeast Asia. Both predictors are closely related to feeding practices, especially the initiation and frequency. It was shown that the initiation of complementary feeding, which was carried out properly, namely at the age of 6-8 months, was able to significantly reduce the odds of stunting by 69%^(25)^. However, when the minimum meal frequency is not met, the risk of stunting increases significantly by 82%^(27)^. Based on the WHO guidelines^(47)^, to get the energy intake needed, the minimum meal frequency must be highly adjusted to the age of child, which is two times for breastfed infants aged 6-8 months, three times for breastfed children aged 9-23 months, and four times for non-breastfed children aged 6-23 months. Therefore, we consider that for a child’s nutrition to be fulfilled, whether the child is still breastfeeding and/or has entered the complementary feeding phase, feeding practices must be carried out in a timely manner and according to age.

### Poor Quality Foods

Furthermore, another causal factor that is also related to feeding practices is low food quality. Of the two predictors classified in this factor, low micronutrient quality is the predictor that appears to provide the greatest increased risk of causing stunting, which is equal to 13%^(33)^. Although few studies have reported it, it was identified that vitamin D^(19)^, iodine^(33)^, iron^(28)^, and vitamin A^(31,33)^ are micronutrients associated with stunting in Southeast Asia. This finding is also the same as that of another study on stunting in other middleand low-income countries^(48)^. Despite the increased risk reported not being as large as other predictors, interventions related to increasing micronutrients in children still need to be focused on Southeast Asia.

Then, for one other predictor, dietary diversity seems to reduce the odds of stunting by 83% if the food group given has more than four variations^(19)^. Apart from being able to provide the energy and macronutrients needed, it was explained that the more diverse types of food, it will promote a more diverse gut microbial community, thereby affecting linear growth^(49)^. However, WHO guidelines^(47)^ explained that there are only seven recommended food groups, including grains, roots, and tubers, legumes and nuts, dairy products, flesh foods, eggs, vitamin-A rich fruits and vegetables, and other fruits and vegetables.

### Inadequate Care

Inadequate care is the next stunting causal factor and which is closely related to the poor way of caring for children. Specifically for children 0-24 months in the Southeast Asia region, we found that poor care practices were the only predictor of this factor. It was also found that incomplete immunization^(17,22)^ and health care practices carried out by third trimester pregnant women^(26)^ were the two manifestations of this predictor. Regarding the reported odds ratio, it is evident that these two manifestations can increase the odds of stunting events up to two times^(17,26)^.

Apriliana et al. (2022) explained that depression and unreadiness, which are felt by most mothers in dealing with their roles during pregnancy, are the reasons that are often associated with poor health care practices of pregnant women^(50)^. It was explained that, when these reasons are not managed adequately, several hormonal imbalances that affect the health of the fetus and the mother, leading to stunting can very likely occur^(50)^. Then, regarding incomplete immunization, it was explained that the susceptibility of children to many infectious diseases and recurrent infections could increase higher than those who were complete. In addition to causing other stunting predictors, especially infection, which definitely increases the odds or risk of stunting, children with this condition have high levels of cytokines that interfere with their appetite and growth hormone. In connection with all these explanations, we consider that this predictor does not actually cause stunting directly, so the management must be adjusted to the poor practices that occur.

### Infection

Specifically for infection factors, there is only one predictor of stunting found in Southeast Asia, namely enteric infection. Sartika et al. (2021) showed that children with enteric infections, namely diarrhea, would increase the odds of stunting in children up to three times^(17)^. Despite providing a high odds of occurrence, no other studies found in Southeast Asia can support this evidence. In addition, no other factors, which were associated with this infection factor to be able to identify other predictors could be found. Furthermore, specifically the food safety factor, which has a predictor of unsafe food preparation, which uses traditional fuels for cooking, which increases the odds of stunting by 50%^(24)^, can cause another predictor of infection, namely respiratory infections. However, for the evidence, especially on this infection factor to be even stronger, further research that identifies predictors of stunting in Southeast Asia is still urgently needed.

### The Contextual Factor

Regarding the four stunting contextual factors found, there are eight significant predictors of stunting. Of the eight predictors, the mother’s employment, ethnicity, and population density are the three predictors that have been shown to provide the greatest increased risk of stunting among other predictors. Sequentially, each of these predictors represents political economy, society and culture, and water, sanitation, and environment factors. Then, specifically on health and health care factors, we review that all studies that support the identified predictors do not analyze the risk or odds of the predictors, so the measure of association with stunting events cannot be clearly known. With these findings, we suggest that further studies are urgently needed to be able to identify in more detail the contextual factors of stunting and their predictors in Southeast Asia.

### Limitations of the Study

There are some limitations found in this review, namely we limit the search for articles in English only, so there may be other articles in national languages that are relevant but cannot be reviewed. Then, not all countries in Southeast Asia provide the articles we need according to our inclusion criteria. However, with the level of socioeconomic status that tends to be the same in each country, the findings of this stunting predictor can represent countries in Southeast Asia.

### Contribution to the Public Health Policy

In reducing stunting, national, and international governments are dominantly focused on nutrition interventions alone. Even though nutritional intervention is not the main key, it is necessary to know other factors first that are in accordance with the causative factors so that they can provide appropriate interventions. With this review, the predictor of factors found can be taken into consideration by policy makers in Southeast Asia in making intervention policies related to stunting, so that interventions and problems found can be resolved. We also recommend that it is necessary to develop integrated health promotion, prevention and intervention in an integrated manner using a multi-sectoral approach to reducing stunting in Southeast Asia involving health care professionals, families, government, and communities. It should always be presented separately from the results.

## CONCLUSION

This scoping review found that of the 13 stunting factors identified using the WHO stunting framework, there are 34 important predictors that must be considered to address the incidence of stunting in children aged 0-24 months in Southeast Asia. Our findings underscore that while Southeast Asian countries should give more focus to maternal conditions, especially before and during pregnancy, and to the suitability of children feeding practice from the time they are born, the development of interventions must remain multi-sectoral and comprehensive. Health promotion and prevention strategies that take an integrated approach and involve health workers, families, governments and communities are essential to effectively address this complex issue.
